# Foreign Accent Syndrome, a Rare Presentation of Schizophrenia in a 34-Year-Old African American Female: A Case Report and Literature Review

**DOI:** 10.1155/2016/8073572

**Published:** 2016-01-26

**Authors:** Kenneth Asogwa, Carolina Nisenoff, Jerome Okudo

**Affiliations:** ^1^Richmond University Medical Center, 355 Bard Avenue, Staten Island, NY 10310, USA; ^2^University of Texas School of Public Health, 1200 Pressler Street, Houston, TX 77030, USA

## Abstract

Foreign Accent Syndrome (FAS) is a rare phenomenon where speech is characterized by a new accent to the patient's native language. More than 100 cases with the syndrome have been published, the majority of which were associated with observed insults of the speech center. Some other cases have been described without identifiable organic brain injury, especially in patients with psychiatric illness. This paper presents a patient with schizophrenia and FAS, without any evidence of organic brain injury. FAS recurred during psychotic exacerbation and did not reverse before transfer to a long-term psychiatric facility. The case is discussed in the context of a brief review of the syndrome.

## 1. Introduction

Foreign Accent Syndrome (FAS) is a rare condition where speech is characterized by a new accent to the patient's native language. This new accent is foreign to both the speaker and the listener [1–4]. It is important to note that the affected patient may never have lived in the country of origin of the new accent [1–4]. There is evidence from the medical literature to suggest that there are three main types of FAS: neurogenic, psychogenic, and mixed. Each of these variants has unique characteristics [5, 6]. There has been an increase in the number of reported FAS cases especially of the neurogenic variety [5]. The patient presented here had a known schizophrenia and psychogenic FAS, a combination for which only few cases have been reported to date in the medical literature.

## 2. Case Report

The patient was a 34-year-old African American US-born single female. At the time of the investigation she was unemployed and lived temporarily with her mother, who had a history of paranoid schizophrenia. The patient was brought to the psychiatry emergency room by ambulance for evaluation of aggression. Upon presentation, the patient described an altercation with her mother's landlady, hitting her numerous times in the face with a closed fist. The patient started the altercation because she felt that the landlady practiced voodoo and had cursed her, causing her hair to fall off. She described an overwhelming rage prior to the physical assault. The patient did not show any remorse for her actions: “I hate her,” “I did the right things,” and “She is evil” are examples of statements made by the patient. Collateral information from the patient's mother revealed that the patient had not been compliant with her medications. She refused to follow up with outpatient care after the last inpatient admission ten months previously. The patient denied auditory hallucinations but appeared to be internally preoccupied. She denied visual and tactile hallucinations, thought insertion, and thought broadcasting. She reported an unchanged pattern of sleep and appetite, which she described to be “good.” Prior to this episode, the patient had been under economic and emotional stress. She lost her job as a nurse aide five months earlier and had not been able to secure another job since then. The patient broke up with her fiancée ten months previously after cutting her fiancée's stepfather's face following verbal altercation. There was no symptom suggestive of mania, seizure disorder, head trauma, loss of consciousness, cerebrovascular accident, Parkinson's disease, anxiety, or other organic brain disorder. The patient denied use of nicotine, alcohol, and other psychoactive substances currently or in the past. The patient had her first inpatient psychiatry admission for acute exacerbation of paranoid schizophrenia and FAS ten months earlier. She was then treated with risperidone tablets with improvement including change in accent on discharge, when she was less psychotic. The patient had a family history of sickle cell disease (brother had sickle cell disease) and schizophrenia (brother, mother, and uncle had schizophrenia). Birth and developmental history were unremarkable. There were no reported behavioral or learning disabilities, and the patient denied being a victim of emotional, physical, or sexual abuse. She had some college education and worked as a nurse aide up till five months prior to presentation to the hospital.

Mental status examination showed a middle-aged, well-groomed, dark-haired woman with poor eye contact and in no apparent distress. The patient was tangential and preoccupied with “voodoo” and was deeply paranoid of her neighbors and her mother's landlady. Auditory hallucinations were not elicited, and she denied suicidal ideation at the time of evaluation. The patient continued to endorse homicidal ideation towards her mother's landlady. She was awake, alert, orientated in time, place, and person, and attentive and had good concentration but had poor impulse control. Her insight and judgment were impaired. Immediate recall and short- and long-term memory were intact.

The physical examination of the patient showed no significant pathological findings. Laboratory investigations were unremarkable. Electroencephalogram showed no seizure activity. Structural magnetic resonance imaging (MRI) and MR angiography of the head were both unremarkable.

The patient presented with a British-like accent, despite never having lived in Britain, and was therefore investigated by a speech therapist. There was no phonetic problem or grammatical errors, but there was a problem with prosody, including prominence of the pitch of the words and syllables. The patient's pitch range was narrow, as she sparingly expressed any emotions during speech. Her speech was monotonous, hesitant, and of low volume. She substituted “th” for “f” and “w” for “wh” as well as “t” for “d” and “ai” for “ei.”

The patient was diagnosed according to DSM-V criteria with paranoid schizophrenia, chronic condition with acute exacerbation, and in addition FAS. The diagnosis of schizophrenia was made based on disorganized speech, specifically tangentiality, paranoid delusions, and grossly disorganized behavior, which lasted for more than ten months and affected the level of functioning, specifically with regard to interpersonal relations and occupation. This was determined not to be due to illicit drug use or known medical illness.

The patient was discharged from the previous hospitalization ten months earlier with a prescription of disintegrating tablets of risperidone. However, she was not compliant with this compound after discharge and also refused to take it during the second hospital admission described here. The patient agreed to take olanzapine tablets to address her psychosis. She was offered a readily dissolvable formula, as we had concern she was “checking” her medications to spit out after administration. However, the patient refused to take any other medication than conventional olanzapine tablets. The olanzapine serum concentration could not be controlled, because the patient became more delusional and refused further blood work. The patient continued to have psychotic symptoms and her foreign accent remained unchanged. She refused augmentation with any other antipsychotic medication because of paranoia. In the course of treatment, the patient continued to endorse homicidal ideation, paranoid ideation towards her mother's landlady, and still spoke in a British accent. In view of unremitting psychotic symptoms, she was transferred to a long-term psychiatric inpatient facility.

## 3. Discussion

Foreign Accent Syndrome (FAS) is a rare speech pathology that presents with an accent perceived to be different from the native accent by the listener and speaker [1–6]. The first reported case, a patient who had a stroke in the left hemisphere, was published in 1907 by the French neurologist Pierre Marie [7]. Since then more than one hundred reports of this syndrome have been published. There are three main variants of FAS reported in the literature: neurogenic, psychogenic, and mixed [5, 6]. For all the types of FAS, the listener's perception is important and ruling out pareidolia is imperative. The characteristics of FAS may involve changes in the pronunciation of words, syntax, and vocabulary as well as changes in the length of the vowels and tenseness, which in phonology is a particular vowel and/or consonant quality that is phonemically contrastive in many languages including English [2, 3, 8]. Other changes include inappropriate stresses of sentences and words. Accents from different parts of the world have been reported and examples include French, English, German, Swedish, Welsh, Spanish, Chinese, Korean, and Irish accents [2, 3, 9, 10]. While there have been many cases of neurogenic FAS, few cases of psychogenic FAS have been reported [5, 6].

In neurogenic FAS, although the exact mechanism is unclear, there is damage observed using neuroimaging techniques to the central nervous system from a stroke or traumatic brain injury [11–20]. Vascular lesions usually involve the middle cerebral artery and motor speech areas such as precentral and middle frontal gyrus, anterior insular, inferior parietal, and adjacent subcortical regions as well as the cerebellum [11–20]. The internal capsule and basal ganglia may also be affected. It has been shown that prosodic function is controlled bilaterally, with the linguistic aspect being controlled by the cortical area of the left hemisphere [21–23].

Until recently it has been assumed that FAS was not psychogenic. In the psychogenic variant of FAS, there may be an underlying psychological or psychiatric disorder such as psychosis, conversion disorder, bipolar disorder, or schizophrenia with ongoing episodes but without identifiable organic brain lesion deficits in the areas connected with language production and speech articulation, suggesting that various other factors and lesions are implicated [2–6, 9, 24–27]. In the cases of psychosis, the new accent persists throughout the entire episode and may disappear after the psychotic episode subsides. The more severe the psychosis is, the more likely the syndrome remains. Many hypotheses have been postulated for FAS in conjunction with psychoses, which include the activation of positive psychotic symptoms, which may also be the provocateur of the accent, and neural circuitry suppression, which may show intermittent prominence [2, 3].

Finally, in the mixed variant, there may be features of both neurogenic and psychogenic characteristics [5, 6]. In these cases, the cause may primarily be neurogenic and then develop to be psychogenic. Researchers have described a loss of identity and the effort of the patient to improve the veracity of the accent as the patient evolves into a new identity [5, 6]. The distinction between neurogenic and psychogenic in phonetic or linguistic terms is important, because it may be the case that these FAS patients have different pronunciation characteristics. The former could result from dysarthria or apraxia, while the latter is somehow “put on” or taken on [5, 6].

When considering linguistics in FAS phonetically, it is important to consider segmental or prosodic characteristics. For segmental characteristics, consonants and vowels are affected and are either reduced, made simpler than usual, missing, unarticulated, or broken [28–32]. Many models of prosody exist and there is no consensus theory [29, 30]. Prosody has two main functions, which include linguistics and paralinguistics. Prosody has many auditory variables, including voice pitch, sound length, loudness, and timbre and acoustic variables, which include frequency, duration, and intensity. These play a role in the functions (intonation and stress) and features (rhythm, tempo, and loudness) of prosody [28–33]. However, it is imperative to mention that the consideration of change in accent as related to FAS is either segmental (articulation, phonation, and speech coordination) or suprasegmental (speech rhythm, duration, and intonation) [28, 31]. Speakers tend to have distinctive speech rhythms [29]. Specifically our patient had a problem with the intonation component; however other aspects of speech may also give similar effects of communication as those achieved by prosody.

According to an autosegmental-metrical framework four levels of intonation exist: inventory, distribution, realization, and function [29]. FAS speakers differ in all of the levels except for inventory. Changes in intonation may be explained by a deficit in the intonation and speech support systems [29, 30]. Problems in intonation include higher mean pitch, reduced pitch range, inappropriately large and sharp pitch excursions on prominent syllables, unusual terminal falls, and inappropriate use of intonation in conversations, for example, when making statements or asking questions [29]. It remains unanswered whether the levels of intonation are a consequence of a diagnosed speech impediment or an adaptive coping strategy for the impediment in another aspect of speech. However, it has been proven that FAS speakers are knowledgeable about intonation and have the ability to use or alter them if required in circumstances of impairment [29–31]. Our patient took on a British accent. British speakers have an average lower pitch as well as precipitous rise and fall in pitch of vowels [2, 3, 34]. The British pronounce the last syllable quickly for consonants, which is different from Americans. The t//d in US English and r linkage for British English apply as an example [34].

Medical literature has described dysfunction of language in terms of form and structure in schizophrenia. It has been suggested that patients with schizophrenia have lower expressivity and complexity even with the same lexicon. The determination of the clinical position of these patients is based on circumstances associated with angry or happy experiences [35]. It has also been determined that differences in lexicon for happiness and anger, in contrast to other types of emotionality, do exist in patients with schizophrenia when compared to patients without this disorder [35].

It has been argued that, following a neurological insult to the language processing regions of the left hemisphere, there is transmigration of certain language functions from the left to the right hemisphere in patients with schizophrenia, which could cause the right hemisphere to dysfunction [36, 37]. Furthermore, it has been shown that the right middle temporal gyrus is associated with the comprehension of emotional prosody in external speech, and patients with schizophrenia are well known to have difficulty with language function in the right hemisphere [37, 38]. Reports have shown that dysprosody associated with Broca's aphasia needs revision since it is associated with the perception of listeners [39]. Since it has been determined that dysprosody is due to lack of continuity in speech of Broca's aphasics, it makes speech appear more melodic to listeners [39]. FAS is also associated with agrammatism, a deficit in sentence production, which is a symptom of Broca's aphasia. The medical literature is replete with studies presenting patients with schizophrenia and bipolar disorder with difficulties in semantic verbal fluency and word finding when compared to controls [40]. There is a deficit of emotional prosody in patients with schizophrenia and these patients perform poorly compared to controls when asked to repeat words or phrases in a specific emotional tone of voice [40]. This deficit has no relationship with medication or years of formal education [36]. Taken together this may give an explanation to the relationship between schizophrenia, suggested to involve a dysfunction of the left hemisphere, and FAS, a highly selective speech problem, yet leaving the language mainly preserved.

There have been a few schools of thought that do not believe that FAS really is a syndrome of foreign accents. One such school believes that this syndrome depends on the listener and not the patient (speaker); that is, rhythm and pronunciation may have been altered but not the accent [8]. However, this phenomenon is not in line with our patient because she was examined thoroughly by a speech therapist and many physicians and family members opined that the patient did have a change of accent from American to British English.

Reversibility of FAS is an uncommon phenomenon. Most of the cases with FAS persist, but there are a few cases where reversal has occurred following right cerebellar hemorrhage, seizure disorder, and acute episodes of psychosis [1, 17]. In the patient described in this report, there was a history of reversibility of the foreign accent after the index episode of psychosis. Our search of the literature showed that including the case presented here there are currently four case reports on psychosis and FAS published (Table 1) [2, 3]. Among the previously reported cases of FAS, one is notable as episodes of FAS occurred with psychotic exacerbation and disappeared with functional and symptomatic improvement [2, 3]. In our patient functional MRI or positron emission tomography (PET) scanning was not performed. Hence, neurological insult as a cause of the FAS could not totally be ruled out. Our patient spoke with a British accent during psychotic exacerbation, and, with risperidone titration, the psychosis and FAS got better, resembling the first reported case of FAS during psychotic exacerbation [3]. In the previous hospitalization, risperidone was effective in controlling the psychosis with FAS in our patient. In the hospitalization episode described here, patient accepted to take conventional olanzapine tablets after she vehemently refused to take any form of risperidone and oral disintegrating olanzapine. The choice to offer olanzapine to the patient was based on a comparative study of risperidone and olanzapine, which showed that both medications were equally effective for the improvement of positive symptoms and insight; however olanzapine showed superior efficacy with respect to negative symptoms, along with lesser extrapyramidal side effects [41]. Our patient did not show any significant psychiatric improvement while on olanzapine. However, adherence to the medication could not be ascertained, as the patient was found repeatedly pretending to swallow her medications but, in actuality, was hiding the pills in her cheek or mouth.

## 4. Conclusion

Foreign Accent Syndrome (FAS) is still a rare and poorly understood condition, especially the psychogenic variety. While there is substantial literature discussing neurogenic FAS, research is still required to better understand the mechanisms of psychogenic FAS and the relationship between FAS and schizophrenia. Prosody with emphasis on intonation was the major FAS problem with our patient. We will follow up with the psychiatric long-term facility on the reversibility of the patient's accent from British back to American in this admission.

## Figures and Tables

**Table 1:** Case reports of combined foreign accent syndrome and schizophrenia.

Reference	Age (years)	Gender	Type of psychosis	Accent	Language	Course
[3]	65	Man	Schizophrenia	American to British	English	Remission
[2]	30	Man	Schizophrenia	American to Jamaican	English	Remission
	Early 30s	Man	Schizophrenia	American to British	English	Remission
Present report	34	Woman	Schizophrenia	American to British	English	Stable
